# Supplementary material for: Shotgun Metagenomics Reveals Skin Microbiome Composition and Function in Infant Atopic Disease
Source: Allergy. Author manuscript; Available in PMC 2026 Oct 20. (PMC13682535; doi:10.1111/all.70449)
Supplement: Supplementary file 1 [file NIHMS2214389-supplement-Figure_S1.pdf]

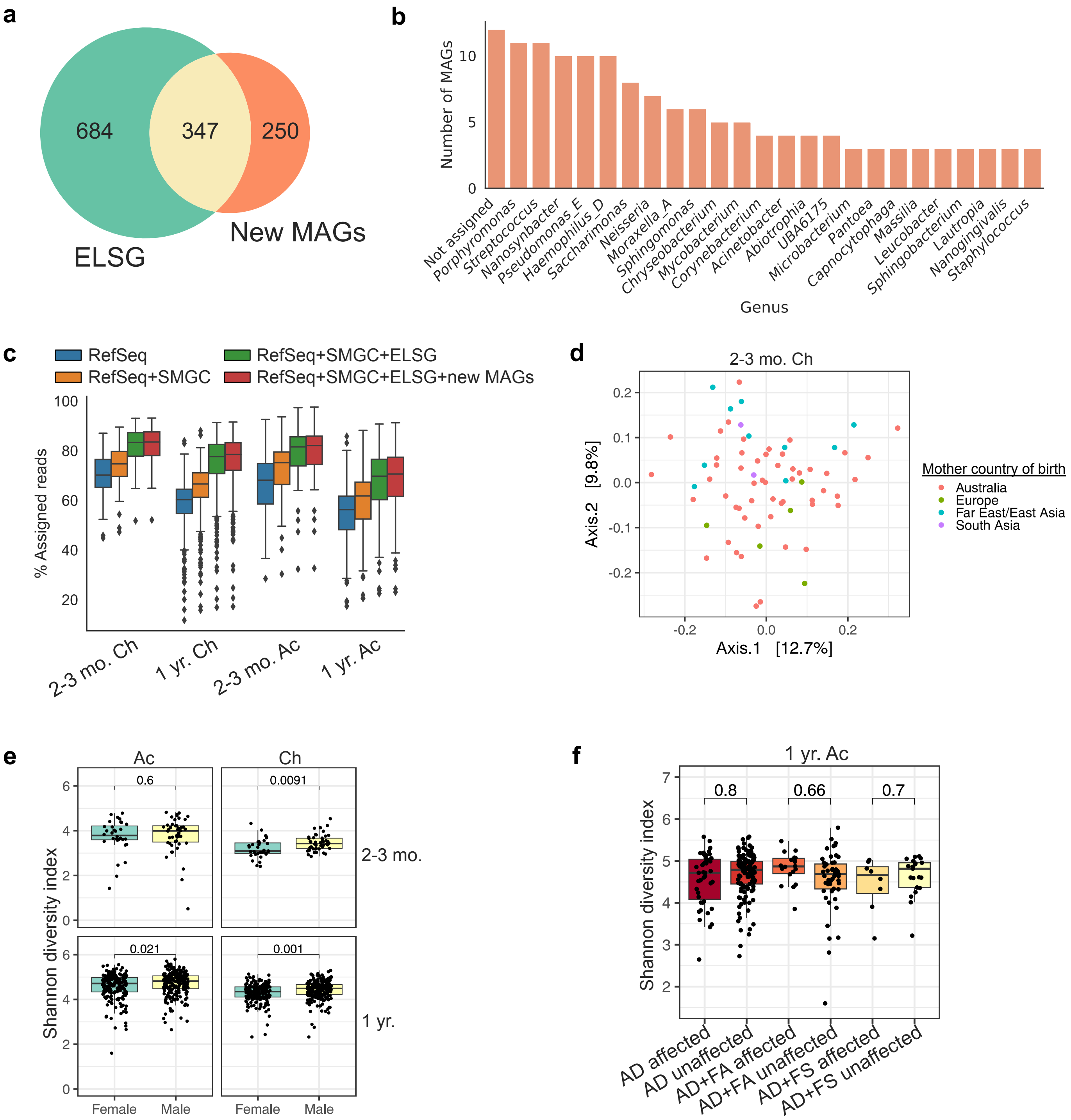
a
684
347
250
ELSG
New MAGs
b
Number of MAGs
Genus
Not assigned
Porphyromonas
Streptococcus
Nanosynbacter
Pseudomonas_E
Haemophilus_D
Saccharimonas
Neisseria
Moraxella_A
Sphingomonas
Chryseobacterium
Mycobacterium
Corynebacterium
Acinetobacter
Abiotrophia
UBA6175
Microbacterium
Pantoea
Capnocytophaga
Massilia
Leucobacter
Sphingobacterium
Lautropia
Nanogingivalis
Staphylococcus
c
RefSeq
RefSeq+SMGC
RefSeq+SMGC+ELSG
RefSeq+SMGC+ELSG+new MAGs
% Assigned reads
2-3 mo. Ch
1 yr. Ch
2-3 mo. Ac
1 yr. Ac
d
2-3 mo. Ch
Axis.2 [9.8%]
Axis.1 [12.7%]
Mother country of birth
Australia
Europe
Far East/East Asia
South Asia
e
Ac
Ch
Shannon diversity index
0.6
0.0091
0.021
0.001
2-3 mo.
1 yr.
Female
Male
f
1 yr. Ac
Shannon diversity index
0.8
0.66
0.7
AD affected
AD unaffected
AD+FA affected
AD+FA unaffected
AD+FS affected
AD+FS unaffected

Figure S1
