# Supplementary material for: Shotgun Metagenomics Reveals Skin Microbiome Composition and Function in Infant Atopic Disease
Source: Allergy. Author manuscript; Available in PMC 2026 Oct 20. (PMC13682535; doi:10.1111/all.70449)
Supplement: Supplementary file 2 [file NIHMS2214389-supplement-Figure_S2.pdf]

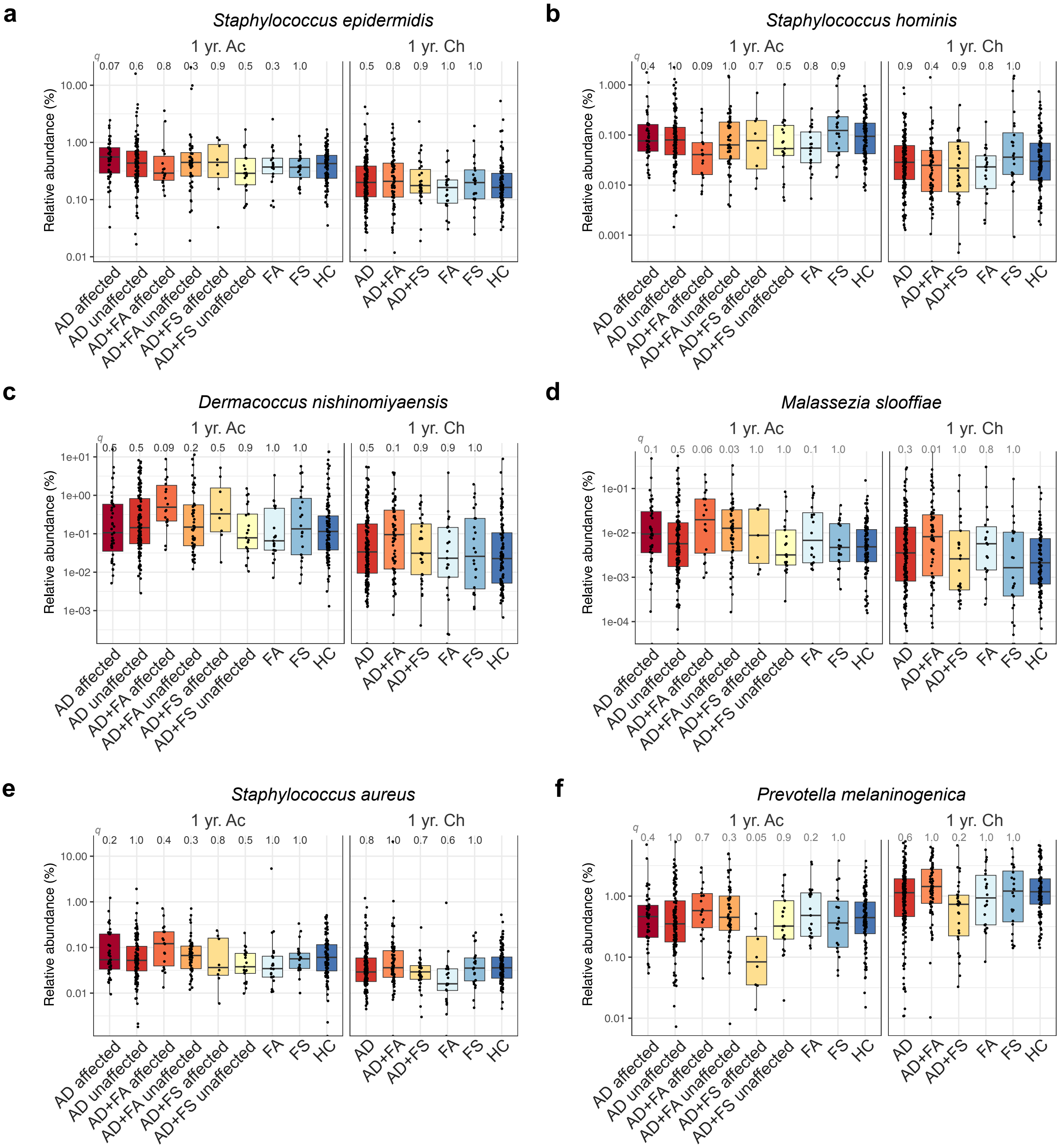

a
Staphylococcus epidermidis
1 yr. Ac
1 yr. Ch
q
0.07 0.6 0.8 0.3 0.9 0.5 0.3 1.0
0.5 0.8 0.9 1.0 1.0
Relative abundance (%)
10.00
1.00
0.10
0.01
AD affected
AD unaffected
AD+FA affected
AD+FA unaffected
AD+FS affected
AD+FS unaffected
FA
FS
HC
AD
AD+FA
AD+FS
FA
FS
HC
b
Staphylococcus hominis
1 yr. Ac
1 yr. Ch
q
0.4 1.0 0.09 1.0 0.7 0.5 0.8 0.9
0.9 0.4 0.9 0.8 1.0
Relative abundance (%)
1.000
0.100
0.010
0.001
AD affected
AD unaffected
AD+FA affected
AD+FA unaffected
AD+FS affected
AD+FS unaffected
FA
FS
HC
AD
AD+FA
AD+FS
FA
FS
HC
c
Dermacoccus nishinomiyaensis
1 yr. Ac
1 yr. Ch
q
0.5 0.5 0.09 0.2 0.5 0.9 1.0 1.0
0.5 0.1 0.9 0.9 1.0
Relative abundance (%)
1e+01
1e+00
1e-01
1e-02
1e-03
AD affected
AD unaffected
AD+FA affected
AD+FA unaffected
AD+FS affected
AD+FS unaffected
FA
FS
HC
AD
AD+FA
AD+FS
FA
FS
HC
d
Malassezia slooffiae
1 yr. Ac
1 yr. Ch
q
0.1 0.5 0.06 0.03 1.0 1.0 0.1 1.0
0.3 0.01 1.0 0.8 1.0
Relative abundance (%)
1e-01
1e-02
1e-03
1e-04
AD affected
AD unaffected
AD+FA affected
AD+FA unaffected
AD+FS affected
AD+FS unaffected
FA
FS
HC
AD
AD+FA
AD+FS
FA
FS
HC
e
Staphylococcus aureus
1 yr. Ac
1 yr. Ch
q
0.2 1.0 0.4 0.3 0.8 0.5 1.0 1.0
0.8 1.0 0.7 0.6 1.0
Relative abundance (%)
10.00
1.00
0.10
0.01
AD affected
AD unaffected
AD+FA affected
AD+FA unaffected
AD+FS affected
AD+FS unaffected
FA
FS
HC
AD
AD+FA
AD+FS
FA
FS
HC
f
Prevotella melaninogenica
1 yr. Ac
1 yr. Ch
q
0.4 1.0 0.7 0.3 0.05 0.9 0.2 1.0
0.6 1.0 0.2 1.0 1.0
Relative abundance (%)
1.00
0.10
0.01
AD affected
AD unaffected
AD+FA affected
AD+FA unaffected
AD+FS affected
AD+FS unaffected
FA
FS
HC
AD
AD+FA
AD+FS
FA
FS
HC

Figure S2
