# Supplementary material for: Shotgun Metagenomics Reveals Skin Microbiome Composition and Function in Infant Atopic Disease
Source: Allergy. Author manuscript; Available in PMC 2026 Oct 20. (PMC13682535; doi:10.1111/all.70449)
Supplement: Supplementary file 3 [file NIHMS2214389-supplement-Figure_S3.pdf]

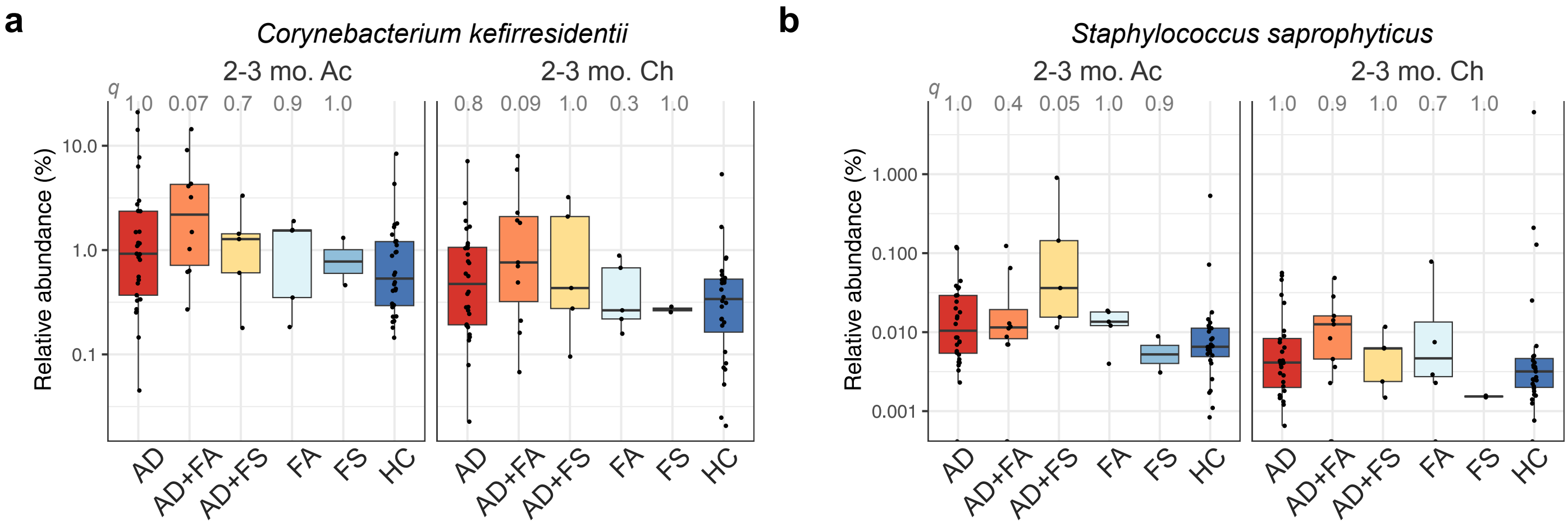
a
Corynebacterium kefirresidentii
2-3 mo. Ac
2-3 mo. Ch
q
1.0 0.07 0.7 0.9 1.0
0.8 0.09 1.0 0.3 1.0
Relative abundance (%)
10.0
1.0
0.1
AD
AD+FA
AD+FS
FA
FS
HC
b
Staphylococcus saprophyticus
2-3 mo. Ac
2-3 mo. Ch
q
1.0 0.4 0.05 1.0 0.9
1.0 0.9 1.0 0.7 1.0
Relative abundance (%)
1.000
0.100
0.010
0.001

Figure S3
