# Supplementary material for: Shotgun Metagenomics Reveals Skin Microbiome Composition and Function in Infant Atopic Disease
Source: Allergy. Author manuscript; Available in PMC 2026 Oct 20. (PMC13682535; doi:10.1111/all.70449)
Supplement: Supplementary file 4 [file NIHMS2214389-supplement-Figure_S4.pdf]

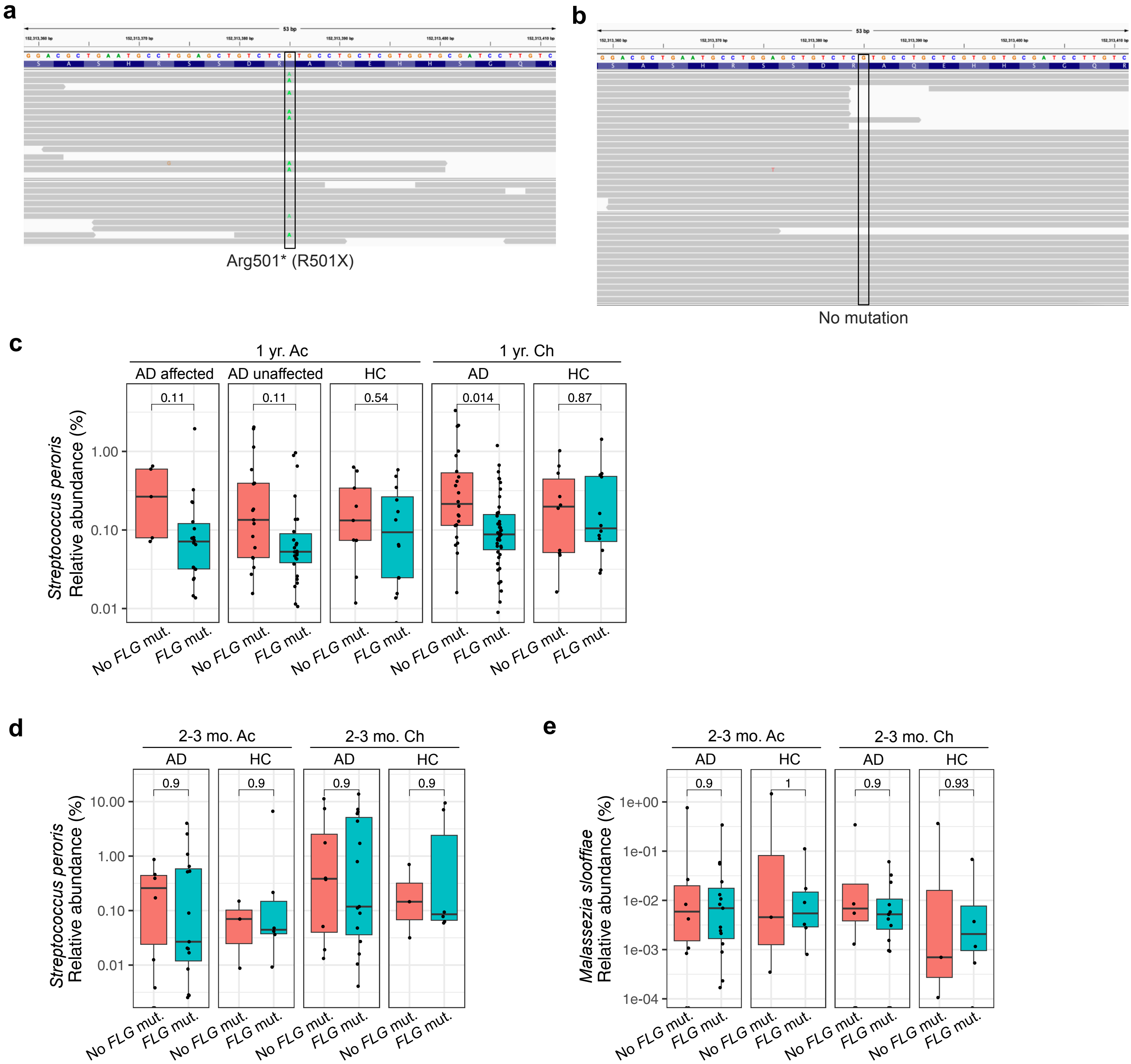
a
Arg501* (R501X)
b
No mutation
c
1 yr. Ac
1 yr. Ch
AD affected
AD unaffected
HC
AD
HC
0.11
0.11
0.54
0.014
0.87
Streptococcus peroris
Relative abundance (%)
1.00
0.10
0.01
No FLG mut.
FLG mut.
d
2-3 mo. Ac
2-3 mo. Ch
AD
HC
AD
HC
0.9
0.9
0.9
0.9
Streptococcus peroris
Relative abundance (%)
10.00
1.00
0.10
0.01
No FLG mut.
FLG mut.
e
2-3 mo. Ac
2-3 mo. Ch
AD
HC
AD
HC
0.9
1
0.9
0.93
Malassezia slooffiae
Relative abundance (%)
1e+00
1e-01
1e-02
1e-03
1e-04
No FLG mut.
FLG mut.

Figure S4
