# Supplementary material for: Shotgun Metagenomics Reveals Skin Microbiome Composition and Function in Infant Atopic Disease
Source: Allergy. Author manuscript; Available in PMC 2026 Oct 20. (PMC13682535; doi:10.1111/all.70449)
Supplement: Supplementary file 5 [file NIHMS2214389-supplement-Figure_S5.pdf]

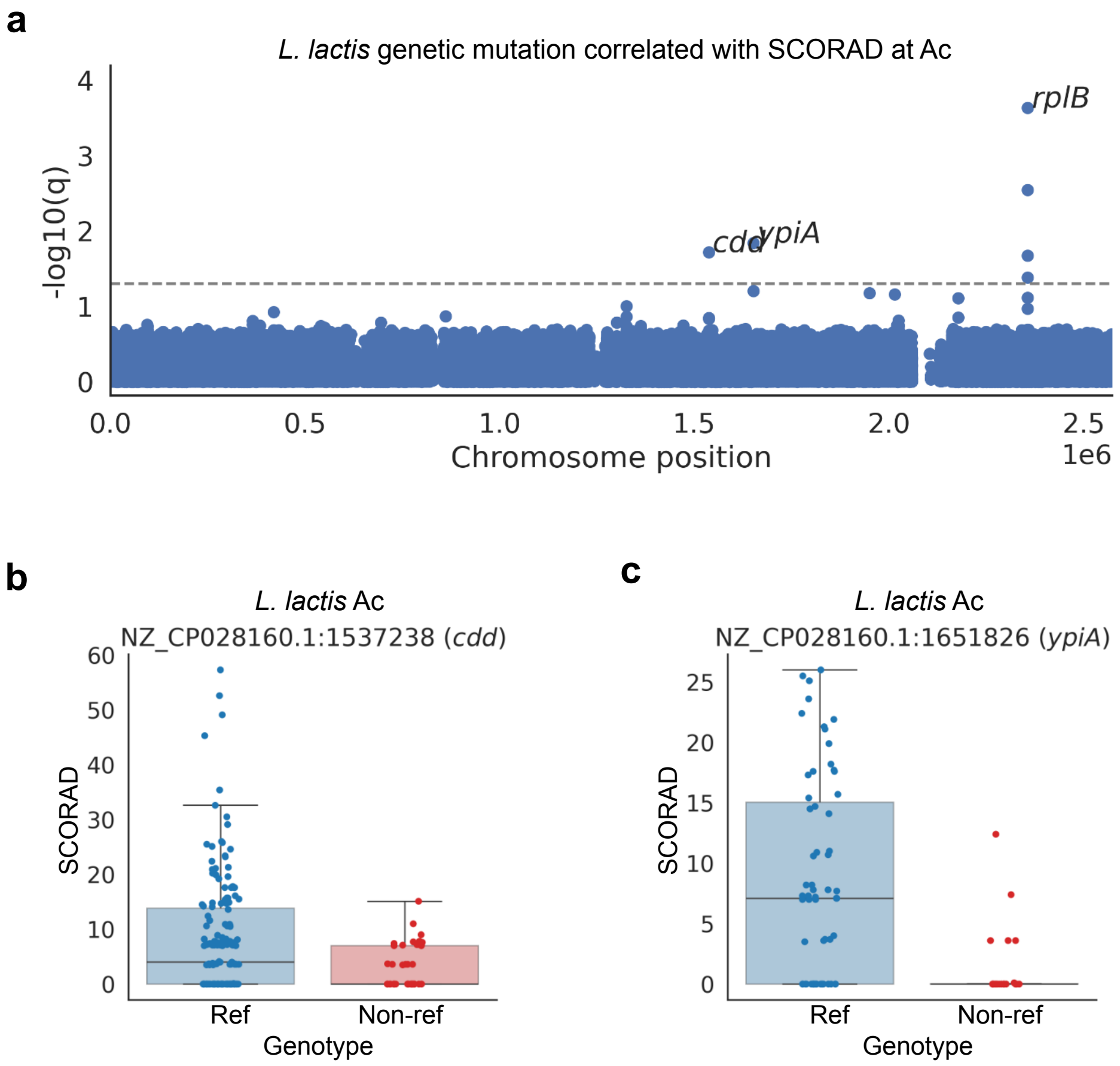
a
L. lactis genetic mutation correlated with SCORAD at Ac
-log10(q)
rplB
cdd
ypiA
Chromosome position
1e6
b
L. lactis Ac
NZ_CP028160.1:1537238 (*cdd*)
SCORAD
Ref
Non-ref
Genotype
c
L. lactis Ac
NZ_CP028160.1:1651826 (*ypiA*)
SCORAD
Ref
Non-ref
Genotype

Figure S5
