# Supplementary material for: Shotgun Metagenomics Reveals Skin Microbiome Composition and Function in Infant Atopic Disease
Source: Allergy. Author manuscript; Available in PMC 2026 Oct 20. (PMC13682535; doi:10.1111/all.70449)
Supplement: Supplementary file 6 [file NIHMS2214389-supplement-Figure_S6.pdf]

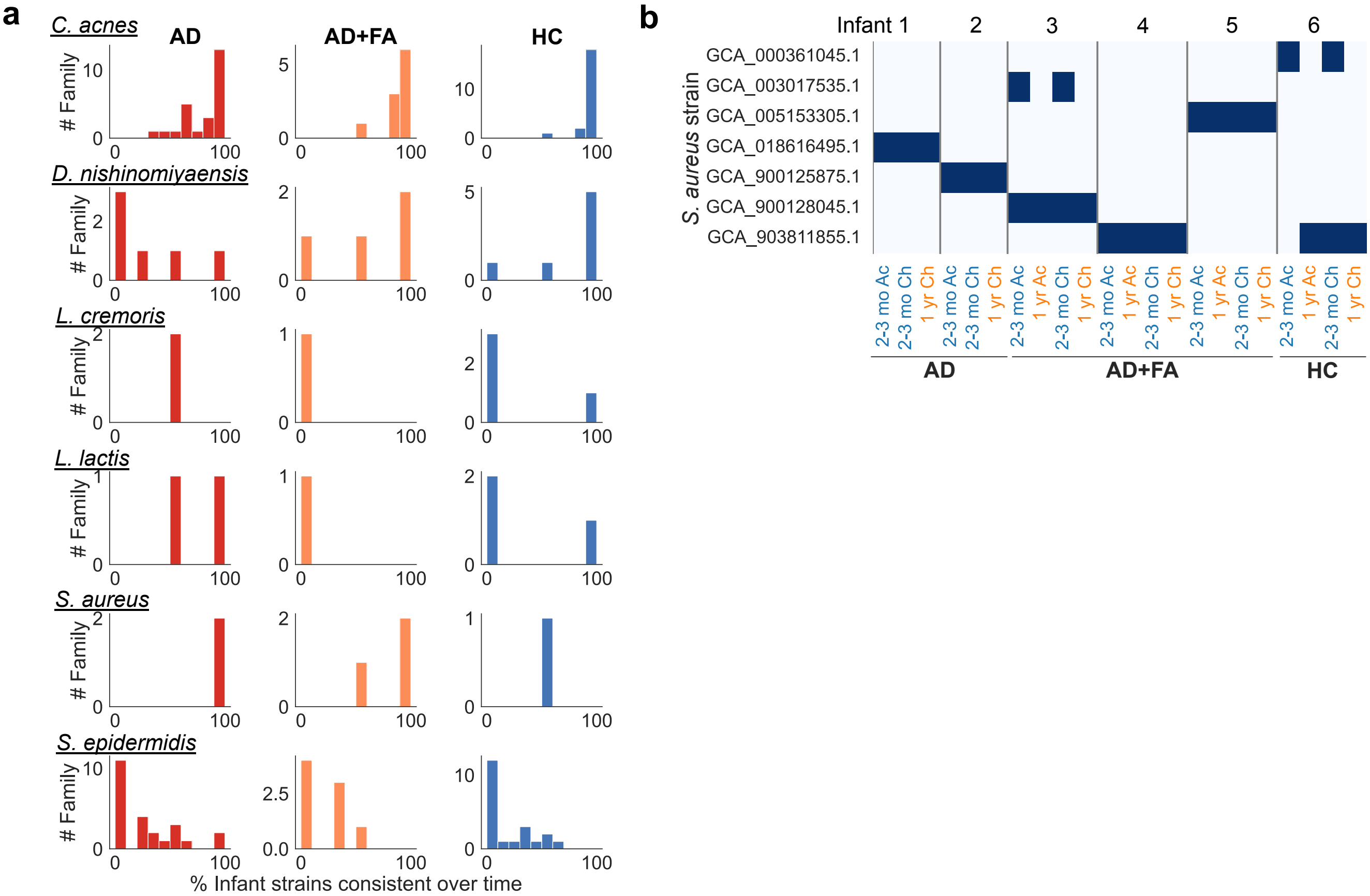
a
C. acnes
D. nishinomiyaensis
L. cremoris
L. lactis
S. aureus
S. epidermidis
AD
AD+FA
HC
Family
% Infant strains consistent over time
b
Infant 1
2
3
4
5
6
S. aureus strain
GCA_000361045.1
GCA_003017535.1
GCA_005153305.1
GCA_018616495.1
GCA_900125875.1
GCA_900128045.1
GCA_903811855.1
2-3 mo Ac
2-3 mo Ch
1 yr Ac
1 yr Ch
AD
AD+FA
HC

Figure S6
